# New Appliances and Things Medical

**Published:** 1906-08-18

**Authors:** 


### NEW APPLIANCES AND THINGS MEDICAL.

[We shall be glad to receive, at our Office, 28 & 29 Southampton Street, Strand, London, W.C., from the manufacturers, specimens of all new preparations and appliances which may be brought out from time to time.]

### THE AUSTRALIAN WINES AND BRANDY.

(Hans Irvine and Company, Dowgate Hill, Cannon Street, London, E.C.)

We have received a case of assorted Australian wines and brandy. The case also contained a bottle of special medicinal wine, the basis of which is Australian burgundy, to which quinine and other bitter tonics have been added. To this wine the name of Irvino has been given. These wines are all produced in Hans Irvine's Great Western Vineyard, of which we have received a descriptive pamphlet. The wines may be roughly divided into red, white, and wines of the port and Madeira type. Of the red wines we have received a half-bottle of Perthonia. This wine is of the type of a French burgundy, full bodied, dry, and of considerable alcoholic strength. It is a thoroughly good wine, but has, as compared with good French burgundy, rather a rougher taste. The Great Western Claret, of which we have also received a sample, is a wine of the Bordeaux type, possessing less body than the Perthonia, but a more delicate bouquet. A serviceable wine for ordinary dinner use is the Red Melbonia, and, for the price of it (which is certainly very cheap), we regard it as very good value. The three white wines which have been sent to us are, in the inverse order of their cost, White Melbonia, White Perthonia, and Great Western Hock. The Great Western Hock is certainly the finest and most delicate wine of these three, and will compare, in our opinion, very favourably with Rhine and Moselle wine. A bottle of Shirazo, or Australian port, has been submitted to us, and considering the price—namely, 30s. per dozen, we regard it as an exceedingly good, light, well-matured wine, eminently suited for invalid or hospital use. The wine to which the name Muscatel has been given is a sweet wine, having a very strongly developed muscat taste. The tonic wine we have referred to above, and there now remains for consideration what we regard as the most important sample of the series—namely, brandy.

There can be no doubt that brandy possesses, by virtue of its compound ether contents, and especially on account of its œnanthic ether, properties as a medicinal stimulant not possessed by any alcoholic solution of the same strength, nor, as we know at present, by any other alcoholic drink. We are not believers in chemical standards, but regard it as most important that no spirit other than that distilled from grape juice should be designated brandy. We have no hesitation in saying that we have formed an exceedingly favourable opinion of this brandy, and regard it as quite suited for invalid use. One of the disadvantages of brandy, at any rate from the point of hospital practice, is its expense, and we therefore welcome the product before us, as its exceedingly moderate price renders it likely to supply a very considerable want.

